# Management of MS Patients Treated With Daclizumab – a Case Series of 267 Patients

**DOI:** 10.3389/fneur.2020.00996

**Published:** 2020-09-08

**Authors:** Paulus S. Rommer, Klaus Berger, David Ellenberger, Firas Fneish, Alexandra Simbrich, Alexander Stahmann, Uwe K. Zettl

**Affiliations:** ^1^Neuroimmunological Section, Department of Neurology, University of Rostock, Rostock, Germany; ^2^Department of Neurology, Medical University of Vienna, Vienna, Austria; ^3^Institute of Epidemiology and Social Medicine, University of Münster, Münster, Germany; ^4^German MS-Register by the German MS Society, MS Forschungs- und Projektentwicklungs-gGmbH, Hanover, Germany

**Keywords:** multiple sclerosis, multiple sclerosis—drug therapy, daclizumab, side effects, registers

## Abstract

Daclizumab was approved by the FDA and the EMA in 2016 for the treatment of relapsing forms of multiple sclerosis (MS). Cases of severe inflammatory brain disease with fatal outcome led to the withdrawal of approval in Europe and the US on March 2, 2018. Approximately 8,000 patients worldwide received daclizumab, but little is known about the further therapy management of these patients after the withdrawal of daclizumab. The aim of this study is to further analyze therapy management in MS patients after safety warnings and market withdrawal. Data from two registries in Germany, the German MS Registry (GMSR) and REGIMS, were used for this analysis. In total, 267 patients were included in this study. For almost 25% of patients (in the GMSR) daclizumab was the initial treatment. Most common pre-treatments were fingolimod, dimethyl fumarate, and natalizumab; various injectables summed up to 25.9%. The most common follow-up therapies were ocrelizumab and fingolimod. In most patients, follow-up therapies were administered shortly after discontinuation of daclizumab. The wash-out time for subsequent therapies varied between 1.2 and 4.0 months. Warnings and decisions by authorities led to a rapid decline and termination of therapies in both cohorts, indicating that such warnings have an immediate impact on the treatment landscape. Therapies that were started within a short time after the discontinuation of daclizumab were subsequently replaced by other therapies and may be considered as bridging therapies.

## Introduction

Since the approval of interferon beta 25 years ago, the treatment options for multiple sclerosis (MS) have increased significantly. The approved drugs vary in terms of efficacy, administration type and side effect profiles. In 2016, daclizumab was granted approval by the Food and Drug Administration (FDA) (1) and by the European Medicines Agency (EMA) (2) based on results of randomized controlled clinical trials (RCTs) for relapsing forms of MS (3, 4). Daclizumab targets CD25, which is identical to the α subunit of the high-affinity interleukin (IL)-2 receptor, composed of the α, β, and γ subunits. Consequently, IL-2 cannot bind to the receptor, resulting in decreased T-cell activation and inflammation. The concentration of soluble IL-2 increases and binds to the non-high affinity IL-2 receptor consisting of the β and γ subunits. This results in an expansion of natural killer (NK) cells (5) and assumed enhanced regulatory effects (6). This unique mode of action promised a new therapeutic option for MS patients without the risks known from other highly effective treatment options, such as infections and progressive multifocal leukoencephalopathy. Common side effects, e.g., rash, hypersensitivity reactions, anaphylaxis, and elevated liver enzymes, as well as autoimmune hepatitis, liver failure, psoriasis, ischemic colitis and inflammatory bowel disease, have been reported in the clinical trials and in previous literature reports (3, 4, 7).

Due to liver failures and a case of fulminant liver disease (8) after approval, the EMA restricted the use of the drug to patients who did not sufficiently respond to at least two previous disease-modifying treatments (DMTs) or for whom other medications were not suitable. In addition, another review on the risks and benefits was conducted in 2017 (2). Reports of severe inflammatory brain disorders with fatal outcomes (9) were reported and surprising. Meanwhile, Luessi et al. (10) described a case of glial fibrillary acidic protein (GFAP) α immunoglobulin G (IgG)-encephalitis in a MS patient treated with daclizumab. Histopathological findings in seven MS patients with encephalitis treated with daclizumab showed infiltration of lymphocytes, eosinophils, plasma cells, and infiltrated vessels treatment (8).

Following the initial reports, the marketing authorization was withdrawn in Europe (2) and in the United States (1) upon the request of the marketing authorization holder (MAH) early March 2018 (11). The EMA recommended the immediate suspension and recall of daclizumab, and that no new patients should start treatment with daclizumab. Further, the EMA recommended treatment with daclizumab should be discontinued in patients and alternative treatments should be considered. Follow-up for at least 6 months was mandatory (12).

Daclizumab was available for use in Germany from July 2016 to April 2018. Worldwide, ~8,000 patients (13) had been treated with daclizumab. In Germany more than 2,800 patients were treated with daclizumab (14). Little is known about treatment changes in these patients after the withdrawal of the drug.

The aim of this study was to analyze treatment switches of daclizumab-treated MS patients after the drug was withdrawn with a focus on the treatment epochs directly before and after daclizumab medication in two independent registries.

## Subjects and Methods

### Data Sources

The German MS Registry (GMSR) was established by the German MS Society in 2001 to provide comprehensive insights into the status of people with MS (PwMS) in Germany (15, 16). It underwent a major technical and data item revision between 2014 and 2016. It includes detailed information on past and current DMTs (17, 18). Since 2014, 30,239 patients were documented in the MS registry, 26,498 of whom had at least one follow-up documentation since 2016. A subgroup of 10,392 patients had complete DMT documentation with specific treatment intervals.

REGIMS is an immunotherapy registry for the improvement of drug safety in MS therapy within the disease-oriented Competence Network Multiple Sclerosis (Kompetenznetz Multiple Sklerose, KKNMS). Its aim is to monitor and analyze the long-term safety and effectiveness of specific immune therapies in patients with MS and to evaluate therapy utilization, including predictors of therapy switches. Since 2014, more than 1,500 patients have been recruited and followed over time for adverse events by standardized physician documentation and annual patient self-reports.

In 2017, both registries started to cooperate closely. In both registries, disease details and sociodemographic characteristics are documented in the same standardized way. Medication histories as well as current treatment and their changes are recorded at the regular follow-up visits. Nevertheless, since its initiation in 2014, REGIMS focuses mainly on safety aspects, while the GMSR pays particular attention to treatment aspects and sociodemographic data. The GMSR has been registered at the German Clinical Trials Register (DRKS, Deutsches Register Klinischer Studien, No. DRKS00011257). The REGIMS registry has been registered at the German Clinical Trials Register (DRKS, Deutsches Register Klinischer Studien, No. DRKS00007190 and No. DRKS 00007127 [Tysabri part]).

### Methods

**Figure 1:**
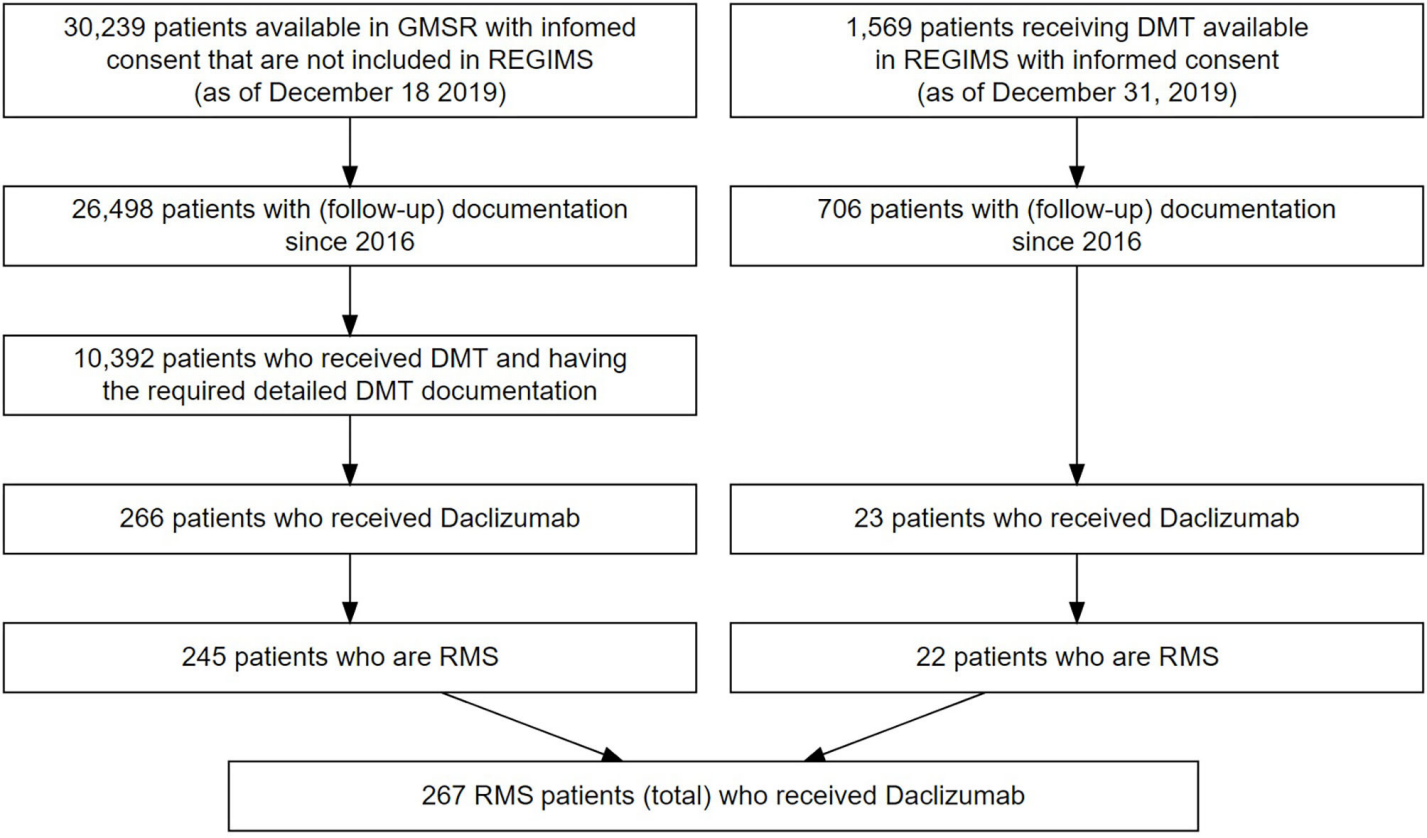
Flowchart of patient selection in the GMSR (left) and from REGIMS (right). 21 patients from the GMSR and one patient from the REGIMS registry suffered from progressive forms of MS and were excluded from analysis.

Inclusion criteria for our study were the approval criteria for daclizumab. Only patients with definite relapsing course of MS were included for analysis. Severity of MS was defined as mild if the score on the Expanded Disability Status Scale (EDSS) at the start of daclizumab treatment was <3, moderate if the EDSS score was ≥3 and <6, and severe if the EDSS score was ≥6. All patients treated with daclizumab with at least one dose were included in the analysis. A total of 245 treated patients in the GMSR and 22 in REGIMS were identified and are included in this analysis (see Figure 1). Some patients are documented in both cohorts but are described under the REGIMS cohort for this study. Data was extracted in December 2019. The treatment duration of each drug was calculated based on the specific start and stop dates, and periods of absence of DMT required confirmation by assessment in clinical visits. The history of administered DMTs varied considerably between patients and the main focus was the identification of drugs used immediately before and after daclizumab, visualized by alluvial style graphs. Annualized relapse rates (ARR) were calculated in subgroups of patients when the exposure times were at least 10 person-years (GMSR only). Analyses and figures were performed with R v3.6.2.

## Results

The patients treated with daclizumab in the GMSR and the REGIMS registry were similar in terms of age at onset, time to diagnosis, duration of treatment and mean EDSS score. Among 245 patients in the GMSR with detailed pre- and post-medication data, 59 patients had no previous DMT and daclizumab was started as *first-line treatment*. This *first-line* patient group was younger, had a shorter disease duration and a lower EDSS score than the second-or-later-line patient group on daclizumab. The average treatment durations with daclizumab were 9.8 months, see Table 1.

**Table 1:** Demographic data of all patients that are part of the German MS registry or of the REGIMS registry and have received daclizumab including 95% confidence intervals for the mean, resp. Clopper-Pearson variants for proportions.

	**First line daclizumab GMSR**	**Second line daclizumab GMSR**	**Third line daclizumab GMSR**	**Fourth line and later daclizumab GMSR**	**GMSR**	**REGIMS**
*N*	59	67	54	65	245	22
Age in 2018, mean	41.8 [38.6–45.0]	41.8 [39.1–44.4]	44.0 [40.8–47.1]	43.7 [41.3–46.0]	42.8 [41.4–44.2]	40.9 [36.8–45.0]
Age onset, mean	31.3 [28.5–34.2]	31.4 [28.9–34.0]	30.3 [27.6–33.1]	28.0 [26.1–29.9]	30.2 [29.0–31.5]	30.4 [26.6–34.2]
Time to diagnosis (years), mean	2.0 [0.5–3.5]	1.1 [0.4–1.7]	3.2 [1.8–4.7]	1.3 [0.4–2.2]	1.9 [1.3–2.5]	1.9 [0.6–3.2]
Disease duration, mean	8.6 [6.1–11.1]	9.3 [7.5–11.0]	12.4 [10.4–14.4]	14.5 [12.6–16.3]	11.3 [10.2–12.3]	8.6 [5.6–11.6]
Treatment dur. (years), mean	0.85 [0.75–0.95]	0.80 [0.70–0.89]	0.79 [0.70–0.88]	0.84 [0.75–0.94]	0.82 [0.77–0.87]	0.89 [0.72–1.06]
Female (%)	67.8% [54.4–79.4]	71.6% [59.3–82.0]	77.8% [11.0–12.0]	80.0% [68.2–88.9]	74.2% [68.3–79.6]	68.2% [45.1,86.1]
Severity						
Mild [EDSS 0.0–2.5]	60.3% [46.6–73.0]	54.8% [41.7–67.5]	44.0% [30.0–58.7]	43.3% [30.6–56.8]	50.9% [44.2–57.5]	61.9% [38.4–81.9]
Moderate [EDSS 3.0–5.5]	22.4% [12.5–35.3]	41.9% [29.5–55.2]	50.0% [35.5–64.5]	41.7% [29.1–55.1]	38.7% [32.4–45.3]	28.6% [11.3–52.2]
Severe [EDSS 6.0–9.5]	17.2% [8.6–29.4]	3.2% [0.4–11.2]	6.0% [1.3–16.5]	15.0% [7.1–26.6]	10.4% [6.8–15.1]	9.5% [1.2–30.4]
EDSS, mean	2.56 [1.97–3.15]	2.58 [2.14–3.02]	2.88 [2.46–3.30]	3.26 [2.79–3.74]	2.82 [2.57–3.06]	2.97 [2.29–3.65]

During the period of daclizumab market authorization, adverse event (AE) reporting was available in REGIMS. AE reporting launched in the GMSR at the end of 2018. In 9 of the 22 patients (40.9%) treated with daclizumab, at least one AE was reported, see Supplemental Table 1. The most frequently reported AEs were infections and infestations (27.3%), musculoskeletal and connective tissue disorders (22.7%) and skin and subcutaneous tissue disorders (18.2%).

### Start and Discontinuation of Daclizumab Treatment During Study Period

**Figure 2:**
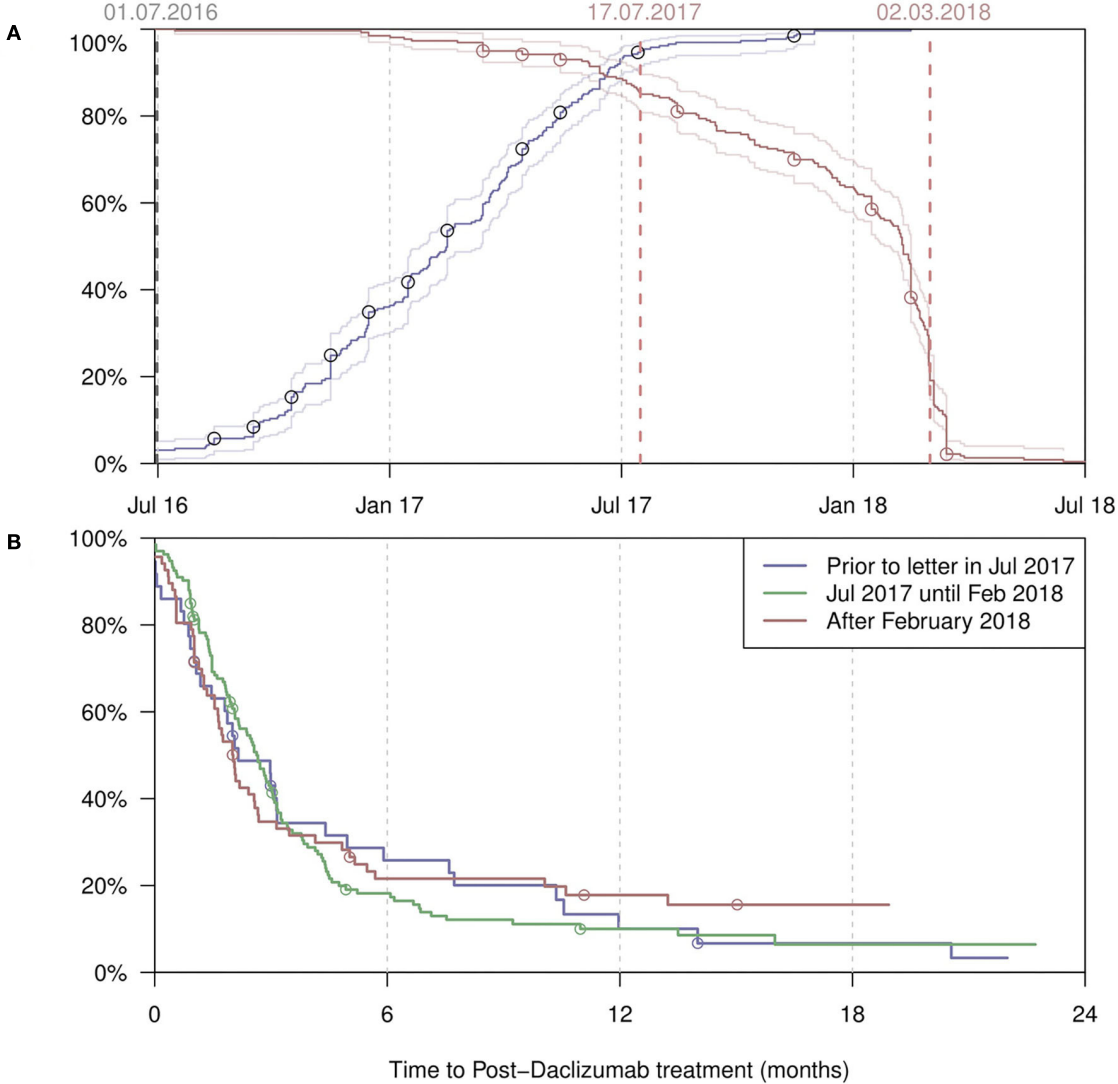
**(A)** Start dates (blue) of all daclizumab treatments, with the percentage of the PwMS having started treatment given on the y-axis. Conversely, end dates (red) with the percentage of the PwMS not having ended treatment given on the y-axis. **(B)** The time from discontinuation of daclizumab till the start of the next treatment, by different time periods when the discontinuation took place. Censoring occurred at last follow-up visit. REGIMS patients (A & B; *n* = 21*) have been marked by circles. One patient from the REGIMS daclizumab cohort (*n* = 22) was excluded from this analysis due to missing exact start and end date of daclizumab treatment.

Figure 2A shows the start and end dates of daclizumab treatment in all patients from both registries. Most treatments were started between July 2016 and June 2017. After the restriction by the EMA in 2017 (2) only a few patients started treatment. Many patients stopped treatment in late summer and fall of 2017. The largest drop occurred between January and April 2018, after the voluntarily withdraw by the MAH (11) and the recommendation by the EMA to immediately suspend and recall daclizumab on 6 March 2018 (12). In Figure 2B, the time interval (or “wash-out period”) between termination of daclizumab treatment and start of a subsequent MS medication is shown, stratified by date of discontinuation. The shortest “switch time” (median 3 months) was observed in patients who stopped daclizumab only after the official withdraw by the MAH.

### Change of Treatment

**Figure 3:**
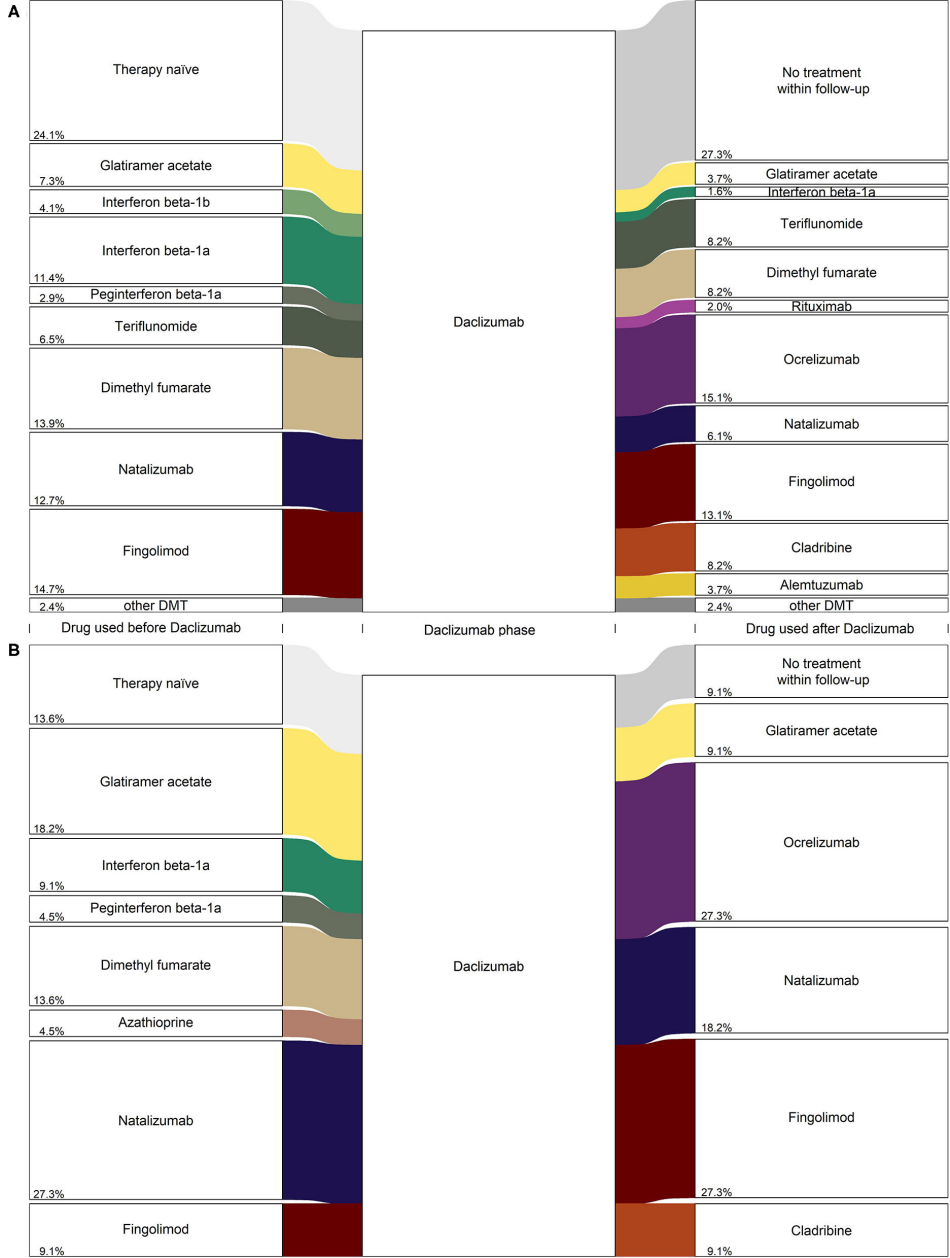
Diagram (alluvial style representation) for daclizumab PwMS from **(A)** the GMSR, *n* = 245 and **(B)** REGIMS, *n* = 22. Only frequencies greater three are displayed for the GMSR data and percentage values are added to the lower left side of the boxes.

Figure 3 shows treatment strategies before and after daclizumab medication, stratified by registry. In the GMSR (Figure 3A) the treatment-naive (24.1%) group was the largest among all single prior treatment groups. Second most frequent prior treatments were fingolimod (14.7%), followed by dimethyl fumarate (13.9%) and natalizumab (12.7%). After withdrawal of daclizumab, the most frequent follow-up treatment was ocrelizumab (15.1%), followed by fingolimod (13.1%) and dimethyl fumarate, teriflunomide, and cladribine (each 8.2%). However, in 27.3% of the daclizumab patients, no subsequent treatment was prescribed until the end of the follow-up period (median 4.7 months).

These findings are similar for daclizumab patients in the REGIMS registry: After the withdrawal of daclizumab, most patients switched to a second-line treatment, with ocrelizumab (27.3%), fingolimod (27.3%), and natalizumab (18.2%) as the most frequent follow-up treatments. For 9.1% of the daclizumab patients from the REGIMS registry, no subsequent treatment until the end of follow-up was documented (Figure 3B).

Regarding the sequence of therapies, only a few patients were switched back to the treatment given prior to daclizumab. Of those patients, 13.3% received natalizumab, 12.5% fingolimod, and 11.1% glatiramer acetate. In contrast, the majority of patients received a new, previously not used therapy (Table 2).

**Table 2:** Descriptions of treatments following daclizumab (GMSR).

**Drug given after daclizumab treatment (GMSR)**	**“Wash out period” in months mean (SD, median)**	**DMT had already been given previously to daclizumab (%)**	**DMT aborted within 6 months following daclizumab (%)**
Teriflunomide	2.6 (5.1, 1.4)	5.0%	31.6%
Rituximab	3.0 (2.5, 3.4)	0.0%	40.0%
Ocrelizumab	4.0 (2.7, 3.8)	0.0%	2.9%
Natalizumab	1.2 (0.7, 1.1)	13.3%	0.0%
Glatiramer acetate	2.8 (3.1, 2.5)	11.1%	12.5%
Fingolimod	2.8 (2.9, 2.4)	12.5%	11.1%
Dimethyl fumarate	2.4 (1.0, 2.4)	10.0%	20.0%
Cladribine	3.9 (1.8, 3.0)	0%	11.8%

Table 2 shows that the wash-out periods after daclizumab treatment and the percentage of switches to a previously unused drug, differed for the various subsequent drugs. The wash-out period was longest for ocrelizumab (4.0 months on average), followed by cladribine with 3.9 months, and was shorter for natalizumab (1.2 months) and dimethyl fumarate with 2.4 months. After daclizumab was withdrawn, most patients received a drug not previously prescribed. Those patients had a shorter wash-out period.

In the GMSR, 38 relapses under daclizumab therapy were reported [annualized relapse rate (ARR): 0.20]. In the last year before treatment with daclizumab, the mean ARR was 0.37. The ARR varied depending on the previous DMT (between 0.13 and 0.73). In the last year prior to treatment with daclizumab, untreated patients had an ARR of 0.35. Patients with a previously terminated DMT, and no treatment in the year prior to daclizumab, also had an ARR of 0.39. In the year after discontinuing daclizumab therapy, the ARR was 0.30.

## Discussion

Daclizumab raised high expectations after it had shown to be efficacious in clinical trials and had demonstrated a unique mode of action. Side effects, including deaths (judged not related to daclizumab) (3, 4, 19), occurred during the clinical trials. However, the number and severity of side effects that emerged after market authorization were surprising and finally led to the withdrawal of daclizumab from the market.

Daclizumab, as a new treatment option, was chosen as *first-line* therapy in about one in four patients, reflecting quick acceptance and/or successful marketing of the new MS therapy in Germany. *First-line* use was more frequent in patients of younger age, with lower MS severity and a shorter disease duration, thus, offering this patients groups new emergent treatments (but with lacking long-time experience).

The initiation of treatment with daclizumab decreased after a case of fulminant liver failure was reported in the second half of 2017 (8), and treatment with daclizumab was stopped promptly after the drug was withdrawn (12). Our analysis suggests that treating physicians reacted very quickly to the published safety concerns. Our study shows that physicians switched their daclizumab-treated patients to a broad range of subsequent therapies, but among daclizumab patients in the GMSR, there was also a considerable proportion with no subsequent drug use in the follow-up time.

Our analysis showed that most subsequent medications had not been used in individual patients prior to daclizumab. Many of these patients switched to newly available drugs instead. The majority of patients who stopped after the official withdrawal were observed to have had follow-up therapy within 3 months. However, at least 20% of patients did not start follow-up treatment within 1 year, which does not indicate an urgency but a general uncertainty instead.

Results from the GMSR data show that 41.4% of the patients were switched to apparently more effective therapies, so-called escalation treatments such as alemtuzumab, cladribine, fingolimod, natalizumab, or ocrelizumab. More than 80% of the daclizumab-treated patients in the REGIMS registry were switched to escalation treatments, and only a small proportion of the REGIMS patients did not receive subsequent treatment until the end of follow-up.

It is noteworthy that only very few patients in the GMSR were switched to baseline therapies like interferons and glatiramer acetate, whereas a comparably high number of patients were switched to therapies that had just entered the market after daclizumab's approval (ocrelizumab and cladribine). While in the case of ocrelizumab physicians have prior experience from rituximab treatments as a fallback medication, it is surprising to see that cladribine had been the choice in more than 8% of the patients in GMSR. This indicates a strong confidence of the treating physicians in the potential of newly approved MS treatment options.

More than one in four daclizumab-treated patients in the GMSR did not receive a subsequent DMT in the follow-up period. The reason for this might be 2-fold: first, the follow-up period with a median of 5 months might have been too short, and second, due to low disease activity or for safety reasons, no subsequent treatment had yet been initiated.

Our study has a number of strengths and limitations. The analyses are based on real-world patient data that were assessed in a highly standardized way. Both registries were operated independently of each other and yielded similar results; thus, they replicated the findings, providing robust results. Among the limitations is the fact that the number of daclizumab-treated patients in REGIMS was rather small and the reasons for the discontinuation of daclizumab were not continuously recorded in the GMSR. However, it has been shown that both the initiation and termination of daclizumab therapy were related in time to the decisions of the authorities. This important observation from the GMSR was confirmed in a second independent registry—REGIMS—which considerably increases the significance of our study. Furthermore, AE reporting during the period of daclizumab market authorization was available for patients in REGIMS. Although the number of these patients was rather small (*n* = 22), at least one AE had been reported for a moderate percentage (40.9%) of this patient group. All AE in this group had been described in prior phase II or III trials, with infections such as nasopharyngitis, upper respiratory tract infections, and cutaneous events being the most frequently reported AE (20, 21).

## Conclusion

In conclusion, new emergent treatment options—such as daclizumab, but also cladribine and ocrelizumab—are integrated in the treatment options of MS directly as they become available even though the data on long-term safety outcomes may be scarce due to the limited time of observation and strict inclusion criteria for patient populations in RCTs.

Warnings and decisions by authorities have a rapid impact on treatment prescriptions. Our results also underline the importance of real-world data for the monitoring of patients on newly approved drugs, which can extend observations on prior RCTs. This is a reason why the GMSR started to roll-out the systematic collection of AE data in late 2018, following its involvement in the EMA workshop on the possible use of MS registries, with the aim: to enable (more) timely identification of safety signals. Real-world data from registries such as the GMSR and REGIMS will rise in importance and provide insights that cannot be provided in this form in pivotal studies.

## Data Availability Statement

Anonymized data will be made available by request from any qualified investigator on terms and conditions of the registries' use-and-access policies and subject to the informed consents of the patients.

## Ethics Statement

The studies involving human participants were reviewed and approved by multiple IRBs. The main ethical approval for GMSR was obtained by the IRB of the medical faculty of the University Würzburg (19 June 2012; 142/12) and the resp. one for REGIMS was obtained by the IRB of the Ruhr University Bochum (15 April 2013; AZ. 4588-13). The patients/participants provided their written informed consent to participate in this study.

## Author Contributions

PR: designed and conceptualized study and drafted the manuscript for intellectual content. KB: major role in the acquisition of data and revised the manuscript for intellectual content. DE: analyzed the data, interpreted the data, and revised the manuscript for intellectual content. FF: analyzed the GMSR-data. ASi: analyzed the REGIMS-data and revised the manuscript for intellectual content. ASt: revised the manuscript for intellectual content and major role in the acquisition of data. UZ: designed and conceptualized study, revised the manuscript for intellectual content, and major role in the acquisition of data. Scientific Advisory Group: Peter Flachenecker, Tim Friede, Judith Haas, Christoph Kleinschnitz, Dieter Pöhlau, Otto Rienhoff designed and conceptualized the GMSR.

## Conflict of Interest

PR received research grants from Biogen, Merck, Roche and Amicus; consultancy or speaker fees from Almirall, Biogen, Celgene, Merck, Roche, Novartis, Sanofi Genzyme and Sandoz. None of them resulted in a conflict of interest regarding the submitted work. KB received a grant from the German Ministry of Education and Research (within the German Competence Net Multiple Sclerosis) plus additional funds from Biogen, all to the University of Muenster for an investigator initiated adverse events register for patients with multiple sclerosis. ASt reports institutional grants for the enhancement of the German MS-Register by Biogen, Celgene, Merck and Novartis, all outside this study. UZ received speaker fees from Alexion, Almirall, Bayer, Biogen, Merck, Novartis, Roche, Sanofi Genzyme and Teva. None of them resulted in a conflict of interest regarding the submitted manuscript. The remaining authors declare that the research was conducted in the absence of any commercial or financial relationships that could be construed as a potential conflict of interest.
