# Supplementary material for: Management of MS Patients Treated With Daclizumab – a Case Series of 267 Patients
Source: Front Neurol. 2020 Sep 8;11:996. doi: 10.3389/fneur.2020.00996 (PMC7506133; doi:10.3389/fneur.2020.00996)
Supplement: Supplementary file 1 [file Data_Sheet_1.PDF]

Supplement Table

AE by System Organ Class	Number of AE	% of patients under risk (n = 22)
Gastrointestinal disorders	3(3F,0M)	13.6%
General disorders and administration site conditions	2(2F,0M)	9.1%
Infections and infestations	6(5F,1M)	27.3%
Injury, poisoning and procedural complications	2(0F,2M)	9.1%
Metabolism and nutrition disorders	2(2F,0M)	9.1%
Musculoskeletal and connective tissue disorders	5(4F,1M)	22.7%
Nervous system disorders	3(3F,0M)	13.6%
Respiratory, thoracic and mediastinal disorders	1(0F,1M)	4.5%
Skin and subcutaneous tissue disorders	4(4F,0M)	14.3%
Surgical and medical procedures	1(1F,0M)	4.5%

Adverse events were coded in MedDRA (Medical Dictionary for Regulatory Activities) and can be coded with several different PT codes. Therefore, absolute numbers of AE reports are lower than shown in this table
AE = adverse event, F = female, M = male, PT = preferred term
Supplemental table: Adverse events reported for daclizumab-treated patients in the REGIMS registry.
